# Applying the Realist Evaluation Approach to the Complex Process of Policy Implementation—The Case of the User Fee Exemption Policy for Cesarean Section in Benin

**DOI:** 10.3389/fpubh.2021.553980

**Published:** 2021-06-08

**Authors:** Jean-Paul Dossou, Sara Van Belle, Bruno Marchal

**Affiliations:** ^1^Centre de Recherche en Reproduction Humaine et en Démographie, Cotonou, Benin; ^2^Complexity and Health Unit, Department of Public Health, Institute of Tropical Medicine, Antwerp, Belgium; ^3^Health Policy Unit, Department of Public Health, Institute of Tropical Medicine, Antwerp, Belgium

**Keywords:** realist methodologies, policy process, implementation, complexity, sub-Saharan Africa, user fees, exemption mechanisms, theory

## Abstract

Realist evaluation is making inroads in the field of health policy and systems research to a large extent because of its good fit with complex issues. Until now, most realist studies focused on evaluating interventions or projects related to health care delivery, organization of health services, education, management, and leadership of health workers in high income countries. With this paper, we apply the realist approach to the study of national health policy implementation in a low resource country. We use the case of the user fee exemption policy for cesarean section in Benin, which we followed up from 2009 to 2018. We report on how realist evaluation can be applied for policy implementation research. We illustrate how we developed the initial programme theory—the starting point of any realist evaluation -, how we designed the study and data collection tools, and how we analyzed the data. For each step, we present current good practices, how we adapted them when needed, the challenges and the lessons learned. We report also on how the dynamic interactions between the central level (the national implementing agency) and the peripheral level (an implementing hospital) shaped the policy implementation. We found that at central level, availability of resources for a given policy is constantly challenged in the competitive national resource allocation arena. Key factors include the political power and the legitimacy of the group supporting the policy. These are influenced by the policy implementation structure, how the actual outputs of the implementation align with promises of the group supporting the policy and consequently how these outputs, the policy and its promoters are perceived by the community. We found that the service providers are key to the implementation, and that they are constrained or influenced by the dependability of the funding, their autonomy, their personal background, and the accountability arrangements. This study can inform the design and implementation of national health policies that involve interactions between central and operational level in other low-income countries.

## Background

The realist methodology, which includes realist synthesis, realist evaluation and realist research, is increasingly acknowledged as an appropriate approach to address complex problems in health policy and system research. Health policy and systems research seeks to “*understand and improve how societies organize themselves in achieving collective health goals, and how different actors interact in the policy and implementation processes to contribute to policy outcomes*” (1). As such, health policy and systems research deals with problems that are mostly complex by nature as they often involve many actors operating in organizations that are embedded in larger systems. Policy actors are often confronted with uncertainty about the issue at hand and the potential solutions, which, to complicate matters further, are context-dependent (1).

The complex nature of many policy issues challenges the ontological, epistemological and methodological foundations of biomedical and clinical research traditions (2). Pawson and Tilley (3) argued that because of their successionist model of causation, these approaches fall short in explaining the complex causal processes underlying policy decisions and policies in general. Realist methodologies provide an alternative model that is rooted in generative causation (4). Compared with the successionist model that attributes outcomes to an intervention on the basis of demonstrating through statistical techniques a constant conjunction between the two, realists seek to identify the mechanisms that underlie change in outcomes, and the influence of context in order to produce a causal explanation. It is increasingly used in health system research (5–10), but little so far in the domain of policy implementation research.

Policy implementation research looks at how governments put policies into effect (11). This field emerged in the 1970s in the United States and has known a number of shifts (12). The first generation adopted a top-down approach, “*a conception of implementation as the hierarchical execution of centrally-defined policy intentions”* [(13), p. 89]. Wildavsky and Pressman (14), seminal authors in this field, reported the implementation failure of a United States federal employment policy for minorities in Oakland. With others authors, like Derthick (15) and Bardach (16), they succeeded in making the case for implementation as a critical step in the policy cycle, but these authors did not focus on explaining the reported challenges (13). In response, the bottom-up approach to policy implementation emerged with authors like Lipsky (17), Elmore (18), Ingram (19), or Hjern and Hull (20). Hjern and Porter [(21), p. 216] analyzed the implementation outcomes of federal policies on manpower training in Germany and Sweden and showed how “*implementation structures (…) formed by the initiative of individuals in relation to a programme*” are important factors. The second generation views implementation as “*the everyday problem-solving strategies of ‘street-level bureaucrats’*” [(13), p. 89], focusing on local agencies more than on central-level officials. A vivid debate between top-down and bottom-up proponents ensued. Third generation authors like Sabatier (22) and Goggin (23) aimed to transcend this debate with “hybrid theories” that combine aspects from top-down and bottom-up approaches. Elmore (24) used forward mapping (starting with implementation and reasoning up to the outcomes) and backward mapping (starting from the outcomes and reasoning back to the decision) to show how actions of both central-level officials and local actors are complementary in shaping policy implementation outcomes.

The fourth generation took a different starting point. Indeed, by the 1990s, emerging market-based policy instruments were a central focus of policy implementation studies in the United States. The concept of multicentricity of power gained prominence, creating room for the fourth generation of policy implementation research with authors like Pierre and Peters (25), Honig (26), Ostrom (27), van Hulst and Yanow (28), and Pfadenhauer et al. (29). This generation is grounded in concepts like neo-institutionalism, institutional arrangements, networks, policy culture, and their complex interactions (12).

Policy making is indeed complex (11). It starts from the moment a policy idea emerges and makes its way to the policy agenda. Policy formulation is a key step in the process of putting the policy into effect, as actors involved or excluded from this process will influence the likelihood that the policy will be adopted by a critical mass of stakeholders (11). Multiple actors at various positions are involved, with specific power positions and interests. Policy actors belong to (in)formal networks and organizations, and often combine different roles and positions in society. Policy implementation is a multi-level process wherein mechanisms are generated at macro-level (federal or national level), meso-level (organizational and local level) and micro-level (individuals and groups within organizations or communities). Context matters as well as feedback loops between mechanisms playing out at different levels. Policy implementation is thus in essence a dynamic process (6, 30). Any change among the actors, the policy instruments, or the political, economic or social dimensions of the policy process can affect implementation outcomes (6, 31). The fourth generation of policy implementation research acknowledges the complex nature of the policy implementation process, which in turn led to particular conceptual and methodological challenges that realist methodologies may be able to address (32).

The objective of this paper is to illustrate the application of the realist evaluation approach to the study of policy implementation gaps, *in casu* related to the user fee exemption policy for cesarean section in Benin. For each step of the study, from eliciting the initial programme theory to formulating the refined programme theory, we will present the current common practices and how we adopted or adapted these to research topic.

## The User Fee Exemption Policy for Cesarean Section in Benin

With the decree N° 2008-730 of 22nd December 2008, President Yayi Boni introduced the user fee exemption policy for cesarean section in Benin. From April 2009 onwards, it was applied by 43 state-owned and non-state-owned hospitals. Pregnant women requiring a cesarean section were exempted from paying for a benefit package including referral within the health district, intravenous infusion, consultation fees, all surgical procedures, drugs and medical consumables, hospitalization, and post-operative check-up. The central implementing agency was assigned the role of checking the volume of cesarean sections declared monthly by the implementing hospitals for eligibility and of reimbursing the implementing hospitals a lump sum of XOF 100,000 (US$196) per cesarean section. We described the agenda setting and policy formulation process, and the significant variations in policy implementation, in a previous paper (33).

### Formulating the Initial Programme Theory

Realist research proceeds in iterative cycles that start and end with the formulation of a programme theory (3, 7). The initial programme theory can be elicited using various avenues. Using “*documentary analysis, interviews and library searches*,” realists can draw upon “*documents, programme architects, practitioners, previous evaluation studies, and social science literature*” (3).

**Figure 1:**
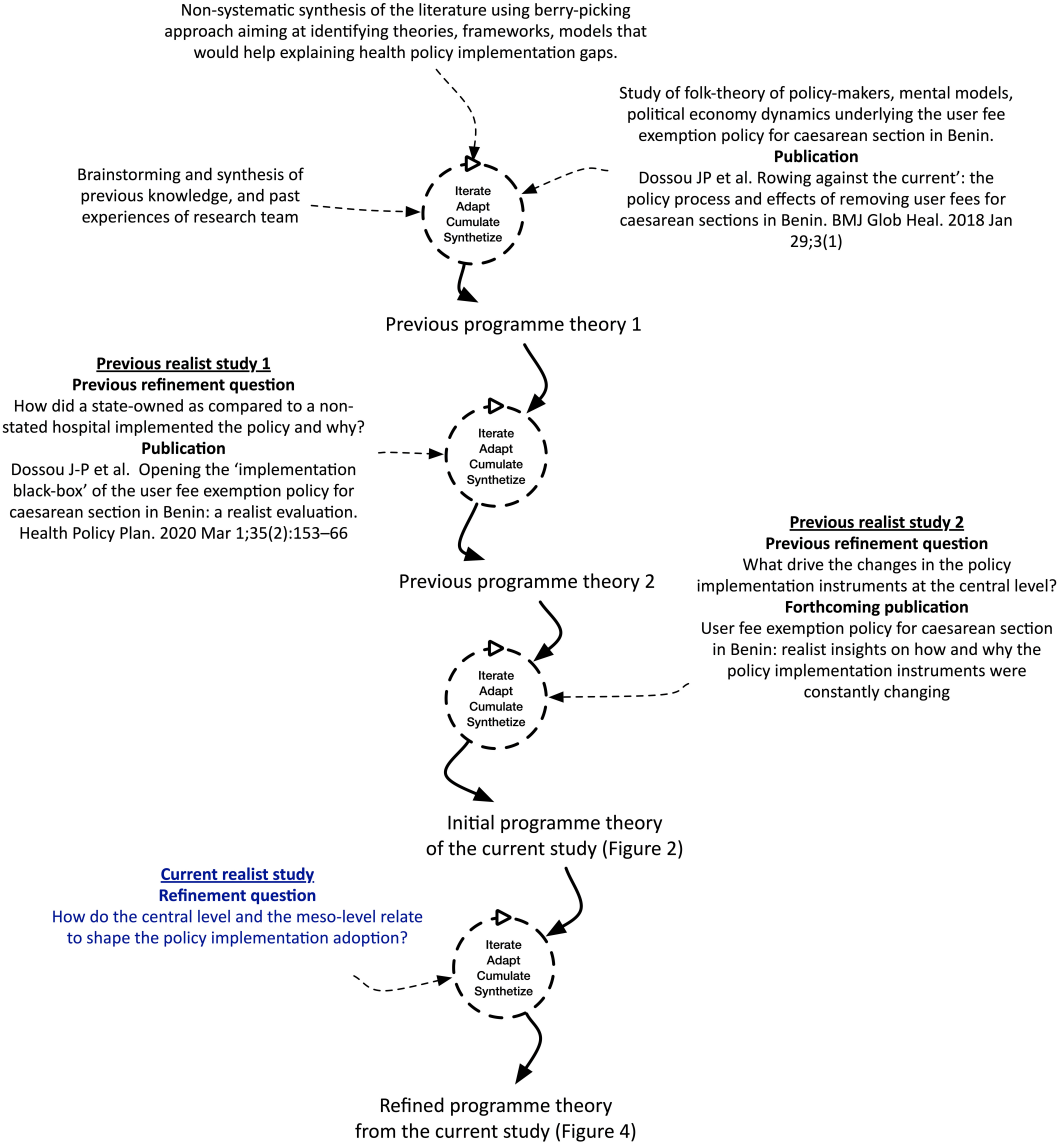
Summary of the research process linking the previous studies with the current one.

**Figure 2:**
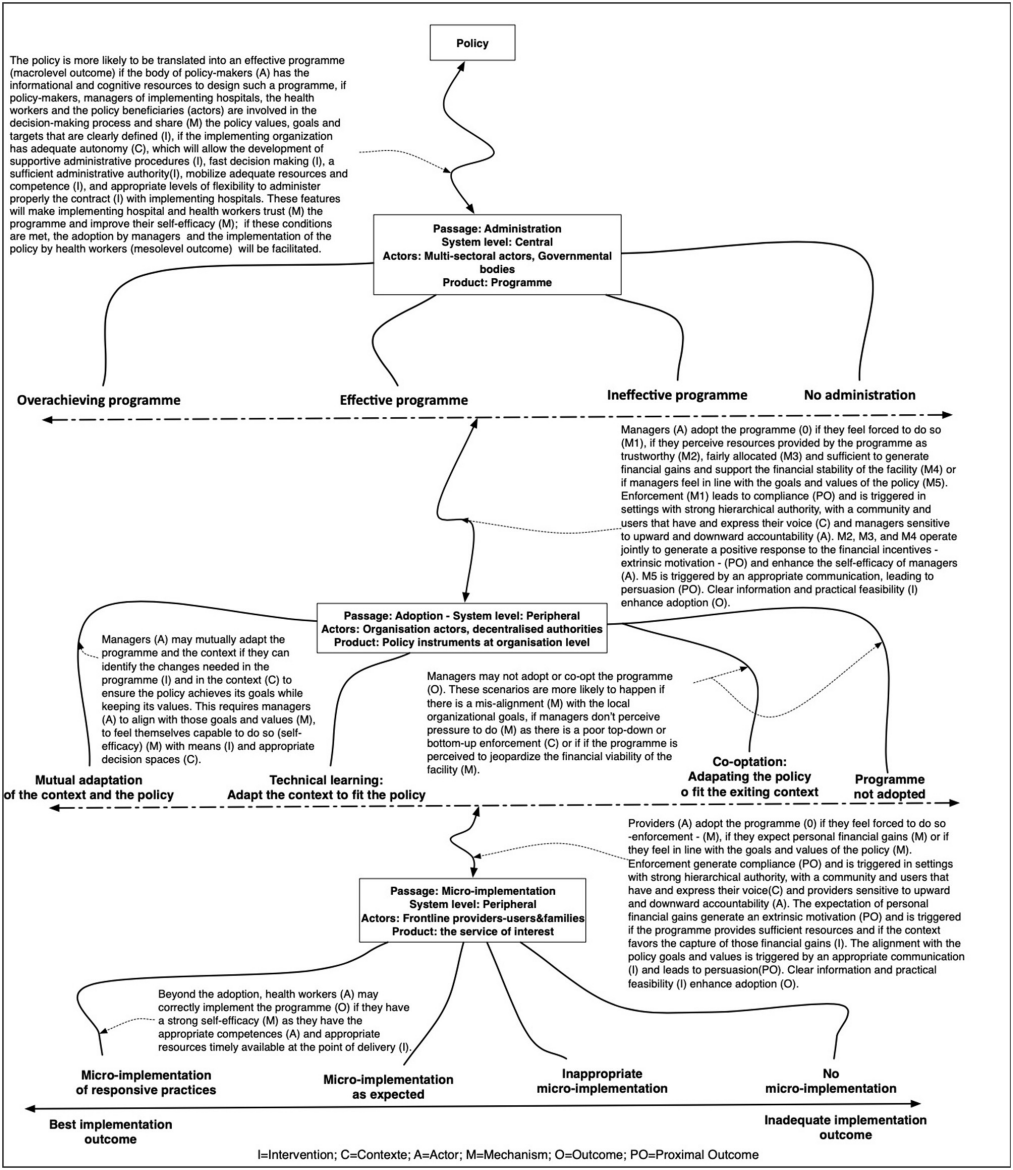
The initial programme theory.

In our study, we built the initial programme theory on the results of a literature review, previous studies on the policy, and personal experience. We started with the literature review aiming at identifying theories, frameworks and models that would help explaining health policy implementation gaps. We specifically searched for publications on maternal health care fee exemption policies in sub-Sahara Africa. We used the “berry-picking” approach (34), combining searching of electronic bibliographic databases, footnote chasing and author searching (35). We found that few publications presented or discussed theories, analytical frameworks or models. In response, we extended our search to the field of public policy and policy implementation, which yielded a number of potential programme theories. In a next step, we organized brain storming sessions with the research team members to think through how the user fee exemption policy would lead to its effects and under which conditions. At that stage, we reviewed policy documents, conducted non-participant observations of policymakers' and implementers' meetings, and conducted interviews with the policymakers and implementers in order to elicit their assumptions and folk-theories on policy implementation, and to identify the political economy dynamics underlying the user fee exemption policy for cesarean section in Benin (33). Finally, we carried out two realist research studies. The first investigated the adoption of the policy by the managers of implementing hospitals and the implementation by health workers. It used a multiple embedded case study design in two hospitals with contrasting policy implementation outputs. It allowed us to formulate a refined programme theory (36) which was tested in a second study (Publication forthcoming). The second study investigated the policy process at the macro- (or national) level, using a single case study design. The unit of analysis was the national level institutional arrangements for the policy implementation from 2009 to 2018. This process summarized in Figure 1 led to the programme theory presented in Figure 2.

For the current study, we explored the importance of the top-down design of the policy, a rather natural phenomenon in Benin's bureaucratically organized health system, in which the central level of the Ministry of Health sets out the policy directions and formulates the actual programme to be implemented by the health districts and hospitals. We focused on the interaction between the central level (arrangements governing the implementing agency) and the managers of the peripheral level (the implementing hospital), considering the dynamic nature of the implementation and the changes in context conditions and over time. We further explored the role of the contractual relationship between the national implementing agency and the implementing hospitals, the financial flows, and the conditions of enforcement. In this study, for clarity we used the terms “central level” and “peripheral level” as they describe better the field of this research as compared to “macro-level” and the “meso-level,” respectively.

### Study Design

In realist research, the study design follows from the initial programme theory and the research question (3). In our study, we adopted a multiple embedded case study design (37). We defined the case as the actual implementation of the user fee exemption policy for cesarean section. We focused on two units of analysis (Table 1), which we followed from 2009 to 2018. The first unit of analysis is located at central level: it is the set of institutional arrangements governing the *Agence nationale de la gratuité de la césarienne*, hereafter called “the implementing agency,” which is in charge of the implementation of the policy. The second unit of analysis is situated at peripheral level and is the implementation of the policy in a state-owned district hospital (hereafter referred to as “the hospital”) with a focus on the hospital managers who play a critical role in the policy implementation. The hospital is a district-level hospital with around 120 beds. It operates an emergency, internal medicine, surgery, pediatric and maternity ward, a pharmacy, a medical imaging unit, and a laboratory with blood transfusion capacity. The maternity ward provides a complete package of emergency obstetric and neonatal care. More recently, an intensive care, a neonatal care, an ophthalmology and a physiotherapy unit were opened. It serves a district with a mix of urban and rural zones, with a population that grew from 264,000 inhabitants in 2005 to about 385,500 inhabitants in 2018. We selected this hospital purposively in function of availability and quality of data for the period of interest on the dimensions of the initial programme theory.

**Table 1:** General characteristics of the units of analysis.

Official name	“*Agence nationale de la gratuité de la césarienne*”	District hospital (name removed) hereafter called the hospital
Legal nature of the organization	Offices with social, cultural and scientific character under the law N° 94-009 of the 28th of July 1994; under the tutorship of the Ministry of Health	Organization with a legal personality and a managerial autonomy under the administrative and technical tutorship of the Ministry of Health
Location	Cotonou, economic capital city located in the south of the country	South of the country
Level	National	District
Date of creation	2009	2001
Role in the policy implementation	According to the decree N° 2009-096 of the 30th March 2009: Ensure the implementation of the policy by designing the implementation strategy, planning, monitoring, and evaluating implementation activities, mobilize the financial resources to ensure the free provision of cesarean section in the state-owned and the non-state-owned facilities.	Ensure the delivery of the cesarean section free of charge to all the women in need who use its services and qualify to benefit from the service; submit financial claims and provide all the administrative details required to monitor and evaluate the policy in its district of service.

### Data Collection Methods

Realist evaluation is method neutral (3): realists set out to collect any data enabling the “testing” of the initial programme theory. In our study, we identified three categories of data: data required to (i) assess the outcome (see Table 2); (ii) describe the case (see Table 3); and (iii) explore the reasonings of the actors in order to identify the mechanisms (see Table 4). Data were collected in three rounds (2012, 2016, and 2018). We present the definition, the purpose and the links to the programme theory as well as the data collection and preliminary analysis strategies in Tables 2–4.

**Table 2:** Tools to assess the policy outcome at various levels: definition, purposes, link with the programme theory, data collection technics, and preliminary analysis strategies.

**Tools, definition, purpose, and link with the programme theory**		**Data collect technics and preliminary analysis strategies**
**Implementation outcome at national level: finances by cesarean section ratio**		Data were collected through document review and semi-structured interviews with key informants from the Agency. Data on the financial resources allocated to the policy were extracted from the administrative records of the ministry of health and triangulated with key informants in charge of budgets at the ministry of health.
	**Definition**	
	Ratio obtained by dividing the total amount of financial resources allocated to the policy in the annual government budget over the validated number of cesarean sections performed every year in all the accredited implementing hospitals between 2009 and 2018.	
	**Purpose and link with the programme theory**	
	This is a measurement of a critical output of the implementation processes happening at the macro or national level of the system in terms of alignment of the resources provided with the number of cesarean sections performed in implementing facilities. Making explicit this ratio and investigating the causal mechanisms underlying its changes overtime makes it possible to test and refine the programme theory.	
**Implementation outcome at hospital level: cesarean section kit completeness index**		This is a quantitative data extraction tool filled by two managers of the pharmacy and by the principal investigator. Data sources included the pharmacy registers, the decrees issued by the hospital managers to set the content of the kits. A quantitative descriptive analysis was carried out.
	**Definition**	
	This a proportion (formulated in percentage) with the following parameters: Numerator: number of items actually provided free of charge based on the decision issued by managers of the hospital, for each year from 2009 to 2018. Denominator: total number of items in the official cesarean section kit issued in 2009.	
	**Purpose and link with the programme theory**	
	The content of the cesarean section kit is a proxy of the resources allocated by the hospital managers to the care providers gynecologists and surgeons, midwives and anesthesiologist in order to deliver the cesarean section free of charge. It allows to track the outputs of the policy implementation at the level of the hospital managers over time.	

**Table 3:** Tools to describe the policy in its context: definition, purpose, link with the programme theory, data collect technics, and preliminary analysis strategies.

**Tools, definition, purpose, and link with the programme theory**		**Data collect technics and preliminary analysis strategies**
**Timeline of the key policy events at national and hospital levels**		Data at national level were collected by reviewing the policy documents, the report of previous research on the policy, press clips, and by conducting (in)formal interviews with key informants from the implementing agency, the ministry of health and the hospital. Data at hospital level were collected using document review and semi-structured interviews with managers of the hospital (the last two directors, the head of the financing department and the focal point of the policy within the management team in 2018) and other intermediary ward managers (the gynecologist, the lead midwife, the lead of the surgery ward and a pharmacy manager). At both levels, data were completed by the personal records of the principal investigator who is monitoring the implementation of this policy since 2012.
	**Definition**	
	This is a record of all the key changes in the content, the context, the actors or the processes of the policy at the national level and at the level of the hospital. It includes initial features and changes in the package of the benefit, the definition of the target, the changes in the reimbursement process, changes in the leadership of the policy and national and hospital level, the main events in the functioning of the hospital including in the pricing of various services, the recruitments and staff motivation policies etc.	
	**Purpose and link with the programme theory**	
	This tool is grounded on the policy triangle framework (38). It allows the collection of critical details to perform a thick description of the policy over time. The initial programme theory mentions the critical role of actors in shaping the policy implementing output at various level. This tool allows the mapping of the actors and provides basic details about their characteristics.	
**Financial flow tracking tool**		Data were collected from the administrative records of the implementing hospital by the principal investigator working together with key members of the management team of the hospital closely involved in the management policy administrative and financial operations in the hospital.
	**Definition**	
	This tool collected data on the timing and the content of the financial claims issued by the implementing hospital, the effectivity and the timing of the claim's verification activities performed by the implementing agency, the amount and the timing of the financial resources transferred from the implementing agency to the implementing hospital, the monitoring and supervision interventions of the Agency in the Hospital. This tool includes captures also data related to the total income expected by the hospital from the policy, the actual income received from the policy, the delay incurred before the reimbursement, the total income of the maternity ward, the total expected income of the hospital.	
	**Purpose and link with the programme theory**	
	This tool collects details on how financial resources flow between the national level and the hospital level; and the set of activities related to it. The initial programme theory presents the financial resources, their amount and timing as critical elements that contributes in shaping the policy implementation output at hospital level.	
**Hospital and maternity capacity assessment tool**		These tools were filled through data extraction from hospital medical records, administrative health system information at the health district level, observation of the facility environment and interviews with a manager, the lead midwife and the lead of the surgery ward.
	**Definition**	
	This tool records the availability of infrastructures, equipment, and functions of the hospital and the maternity ward over time from 2012 to 2018. It records also details on the number and the nature of the contractual arrangements with key staffs involved in the provision of maternity care service and cesarean section.	
	**Purpose and link with the programme theory**	
	The initial programme theory presents the capacities of the implementing hospital, the resources available to perform the services as a contextual factor that may that contributes in shaping the policy implementation output at hospital level.	
**Service provision monitoring tool**		
	**Definition**	
	This tool records the number of admissions in the hospital and in the maternity ward, the number of deliveries and the number of cesarean sections provided.	
	**Purpose and link with the programme theory**	
	It contributes to describing the context of the policy implementation in hospitals, by showing trends in the workload of the hospital, providing a proxy of the attractiveness of the hospital and its performance, and by providing details on the proportion of delivery by cesarean section in the hospital and its trend over time.	
**Organizational autonomy assessment tool**		This has been completed by the interviewer through semi-structured interviews with two top managers of the hospital (the past and the present) who ruled the hospital from 2009 to 2018.
	**Definition**	
	We assessed the organizational autonomy of the Agency and of the Hospital using the tool, based on the conceptual framework of organizational autonomy of government agencies (39). For each of the following dimensions or autonomy (management, policy, structural, financial, legal and interventional), a score from 1 (very low) to 4 (very high) is applied based on a predefined statement that best fit with the situation of the agency or of the hospital.	
	**Purpose and link with the programme theory**	
	From the initial programme theory, organizational autonomy appears to be key context factor, that can influence which decision space actors have at different levels and how they use it and interact with the policy. However, the way organizational autonomy operates in practice is not clearly formulated yet in the programme theory and hence get a special interest in this study.	
**Trust dynamics assessment tool**		This has been completed by the interviewer though semi-structured interviews with the two top managers of the hospital who ruled the hospital from 2009 to 2018 and the leaders of the maternity and the surgery wards.
	**Definition**	
	This tool aims to capture changes in the trust between i) the managers, the maternity and surgery leaders toward the implementing agency; ii) the maternity and surgery leaders toward the managers of the hospital, iii) Hospital managers toward the maternity and surgery leaders. We used a scale from −5 to +5 and asked the interviewee to rate to which extent he considers the other party as trustworthy by year from 2009 to 2018.	
	**Purpose and link with the programme theory**	
	From the initial programme theory, trustworthiness of the resources provided by the policy have been clearly identified as a condition for managers and also for providers to adopt the policy. We explore in this realist study the level of trust between various stakeholders and how it operates in relation to the policy outcome over time.	

**Table 4:** Tools to explore the reasonings of actors: definition, purpose, link with the programme theory, data collect technics, and preliminary analysis strategies.

**Tools, definition, purpose, and link with the programme theory**		**Data collect technics and preliminary analysis strategies**
**Interview guide**		
	**Definition** This is a semi-structured interview aimed at document how policymakers and implementers explain the policy implementation output assessed in relation with the case description. This includes elements like their perceptions of the context, their interpretation of the events, and the reasonings that generated their choices. **Purpose and link with the programme theory** This tool produced a thick description that highlights the role of actors, their perceptions of the context, their interpretations of the events, their reasonings and the actual behaviors in relation with relevant political, social and economic features around the critical changes within the policy—all over the period of interest from 2009 to 2018. This provides a set of material further analyzed and triangulated with finding from the other tools to identify the ICAMOs.	A realist interviewing approach has been used. This tool has been administered to five key stakeholders including top and mid-level managers and providers. Those interviews were either done concurrently with the collection of case descriptions or performed after a first round of desk analysis of outcomes and case descriptions.

### Data Analysis

Realist research is characterized by a configurational approach to analysis: starting from assessing the outcome, realists explore the causal pathway that led to these outcomes. Typically, the Context-Mechanism-Outcome heuristic is used to guide the data analysis process (3). The end point of the analysis is a causal statement that explains how an intervention or policy triggered mechanisms among specific actors which led to the observed outcomes in specific context conditions.

In this study, we used the Intervention-Context-Actor-Mechanism-Outcome (ICAMO) heuristic, which aims at making the role of actors more explicit and facilitates a more nuanced distinction between context and intervention (40). We defined mechanisms as “reasoning” of actors interpreting and responding to the “resources” provided by the programme and/or by other interventions in a given context (3).

The analysis started during the field work, whereby the first author actively started to make sense of the data while collecting them, compare initial findings to the initial programme theory and adapting the data collection when needed. Once all data were collected, the qualitative data were imported in NVIVO 12 for Mac and coded. We adopted thematic analysis principles (41) whereby the initial coding tree was based on the key themes of the initial programme theory. This coding tree evolved during the analytical process as new themes were added. We identified the ICAMO configurations by triangulating data from the various sources, using the retroductive approach, in which the researcher starts from the observed events to identify and postulate the conditions without which these events cannot exist (42). Multiple conjectural ICAMOs emerged, which were compared and checked against the data, leading to consolidation of an ICAMO configuration that provided the most plausible explanation of the observed outcomes.

### Refining the Initial Programme Theory

A realist study ends with a refined programme theory, which results from a careful abstraction of the consolidated ICAMO configurations to the level of the initial programme theory, which is then adapted if needed (3).

We present first the findings of our study in the next section and present then the “translation” of the empirical results to the level of the refined programme theory.

## Findings

In this section, we first describe the policy implementation outputs and the practices of policy makers and implementers related to the policy with focus on the two units of analysis. This is followed by a description of the ICAMO configurations we identified.

### The Central and the Peripheral Level Policy Implementation Outputs Over Time

The fee exemption policy set out to reduce the financial barriers to access and use of maternal health services, specifically as related to cesarean sections. At the level of the implementing agency, we found that the amount of financial resources allocated to the policy as well as the actual rate for reimbursing a cesarean section dropped consistently over time (see black and single line in Figure 2).

**Figure 3:**
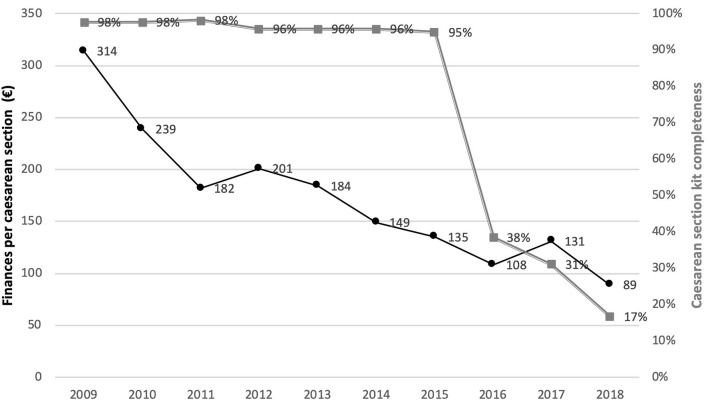
Trend of the financial resources allocated to the policy per cesarean section (at the central level) and cesarean section kit completeness in the implementing hospital (at the peripheral level) between 2009 and 2018.

We used the completeness of the cesarean section kit as a proxy for policy implementation output at the level of the hospital. The cesarean section kit should contain the full set of drugs and consumables that is required for a cesarean section. Under the policy, the kit is free. In practice, women pay for missing drugs and consumables. The more complete the kit, the lower the cost of the cesarean section. At the level of the district hospital, our analysis showed that there were two phases. Between 2009 and 2015, we found the kits were quite complete, but between 2016 and 2018, the kits contained only between 17 and 38% of the items (double gray line on Figure 3).

### Policy Implementation Practices From 2009 to 2015

#### At the Implementing Hospital

Our analysis showed that this hospital was not much used by the community between 2005 and 2009, before the fee exemption policy was introduced. Our respondents mentioned that the community considered the availability and quality of services in the hospital to be sub-optimal. The community members voiced several complaints and exerted pressure that culminated in the period 2008–2009.

In 2009, the management team was led by “M1.” He was dedicated to the hospital's providers, prioritized their well-being, and tended not to question the decisions of the hospital's specialists. We found he perceived the policy as an opportunity to meet the community's demands for better availability of services and quality of care. He considered that the policy is

“*a state policy for vulnerable women; to do otherwise (not adopting the policy), you will be socially frowned upon. For your hierarchy, it is a disobedience and we tried to sustain it for the happiness of the policy targets.” (Semi-structured interview, Hospital manager, Male, 2012)*

From 2009 to 2015, the government reimbursed the hospital on a regular basis. M1 found that the reimbursement process during the initial phase of the policy implementation was fast and easy. He was comforted by the positive feedback he received from the Implementing Agency, who considered the Hospital as a good pupil in terms of policy implementation. When asked about the role of trust in implementing the policy, the hospital managers reported they live in a “*forced trust”* relationship with the state, since they are managing a state-owned hospital. The timing of the reimbursement determined the trust of M1 in the state, and as indicated in Table 2, the trust index of the hospital's managers remained high during this period.

Based on the perceived steady revenue, M1 attracted new gynecologists and surgeons. Contracted by the hospital, the specialists were engaged to work a given number of hours per week; in return, the hospital agreed to pay them a fixed fee of €46 per 24 h and an extra fee linked to the number of surgical procedures they performed. For a cesarean section for instance, this extra fee corresponds to 20% of the €76 fee charged for the surgical act. The number of staff performing cesarean sections in the hospital increased from three in 2008 to eight in 2011. The number of deliveries doubled between 2008 and 2012, and the proportion of delivery by cesarean section increased from 33.3% in 2009 to 69% in 2011. The overall utilization of the hospital increased as the total number of admissions increased from 2.980 in 2008 to 4.839 in 2011.

Several interviewees reported that the community's perceptions about the hospital improved and they found the hospital had become more attractive. As a manager reported:

“*The providers were motivated, they were working well, and the hospital was working. The population was satisfied with this strategy since patients were treated without delay. The fame of the hospital skyrocketed. So, there was a gain for the providers, a gain for us who manage the system and a gain for the population.” (Semi-structured interview, Hospital manager, 2018)*

#### At the Central Level

An important champion of the policy since inception in 2009 was President Yayi Boni (elected in 2006, re-elected in 2011). Several technical staff of the Ministry of Health, health providers and community representatives portrayed the strong personal support from the president as a means to seek popular support for re-election in 2011. This is in line with our finding that prior to his re-election in 2011, the President used the policy as a campaign argument, presenting it as a successful social programme of his first mandate.

At the level of the implementing agency, from 2009 and up to 2013, the terms and obligations of the relationship between the central implementation agency and the hospital were not clearly formulated, and the contract was not signed. In 2013, the Agency introduced a system of contracting implementing facilities in response to reports of hospitals that still charged fees for items that were supposed to be free. The contract enforced the benefit package defined in the Policy decree and specified that the Agency has to reimburse the Hospital within 20 days after the validation of the claims for reimbursement. The contract also stated that in case a hospital is found to charge fees, administrative and criminal sanctions would be raised and the Hospital would be required to pay back these fees. In practice, our data show that from 2014 onwards, important delays were observed in the reimbursement of the Hospital by the Agency. In our interviews, managers of the Hospital declared they were not surprised by this failure, and that this situation reinforced their feeling of being forced to implement the policy. On the other hand, we found that during this period, no procedures were initiated to monitor the fees and that hospitals still charged patients.

#### The ICAMO Configurations for the Period 2009–2015

Related to the intervention, we found that sufficient financial resources were provided regularly at predictable moments (I). The monitoring from the implementing agency (Actors) focused on checking the expenses claimed; the actual user fee removal was not monitored (I). The personal engagement of the president signaled very high political support for the policy (Context). There were no other policy or initiatives with similar goals that needed funding (Context). Ministry of Health staff (A) questioned the relevance and sustainability of the policy but as civil servants, they considered it their duty to comply to governmental policy (O). This was enhanced by their perception that the policy would generate popular support toward the political regime in place and favor its re-election (M). At central level, managers and policy makers trusted the implementing hospital to apply the policy as planned. In this configuration, the implementation resulted in sufficient resources being allocated to the policy at the central level (Outcome).

The policy was introduced in the peripheral implementing hospital at a moment when the hospital managers (Actors) were experiencing a high bottom-up pressure to increase availability and quality of services, and were looking for opportunities to respond to it (Context). They felt the political pressure as a strong constraint; they did not want to be perceived as disobedient civil servants and become socially excluded. The hospital managers perceived the regularity of the reimbursement as a stabilization factor (Mechanism), which reduced their distrust toward the state (Intermediate outcome). They perceived the policy as a double win situation (Mechanism): not only was it an opportunity to comply both with the bottom-up and top-down pressure, but also to improve the financial situation of the hospital and maintain the motivation of their health workers. Managers believed they have enough autonomy to make necessary “positive” adaptations in the policy (Context). As a result, the hospital managers ensured the implementation of the policy (O).

### Policy Implementation Practices From 2016 to 18

#### At the Implementing Agency

Open critique emerged around 2016, when Ministry of Health staff questioned the fee exemption policy (33) and argued for a national health insurance scheme. The latter received considerable support under the presidency of Patrice Talon from 2016 onwards. His first Minister of Health declared in the media that:

“*The free care approach for all is itself a problem. Many Beninese can pay and we offer them free care… The worst point is that, if we continue like this, we will waste so much resources that we will not be able to address and guarantee the fee exemption for those who need it the most.” (Minister of Health of Benin, 2018 in interview with journalists)*

Moreover, the staff of the Ministry of Health in charge of resource allocation reported that despite the correct funding of the policy, women were still paying a significant fees and were expressing low satisfaction with the policy. The staff expressed doubts about the efficiency of the policy and they feared corruption:

“*Mistrust is the problem! Because we realized that the amounts the providers claimed was higher than the services which were provided. There are women who give birth naturally but who are counted as beneficiaries of the policy.” (Semi-structured interview, Manager at the ministry of health, 2018)*

#### At the Hospital

Our analysis found that the delays in reimbursement of the hospital increased. For instance, cesarean sections carried out in August 2016 were reimbursed in May 2018. Up to the end of June 2018, none of the cesarean section performed in 2017 and in 2018 were reimbursed yet.

In 2016, manager M2 replaced M1. During his tenure, the trust index plunged from +2 to −3 (Table 2). According to M2, the arrangement introduced by M1 to attract and maintain specialists created a perverse incentive, generating unnecessary cesarean sections and poorer quality of services:

“*Since providers are paid directly on the hospital budget and paid by percentage (receiving a share of the income generated by their services), the more interventions they do, the higher is the payment they receive. So the service provided is no longer objective, the service provision became driven by personal interests… So, there are cases (of caesarean section) we can avoid, but we do them anyway.”(Semi-structured interview, Hospital manager, 2018)*

Aiming at both lowering the rate of unnecessary cesarean sections and reducing the loss of revenue due to the reimbursement delays, M2 reduced the number of medical staff under contract. She kept only one trusted gynecologist. The number of cesarean sections performed in the hospital decreased by 24% in 2017, as compared to 2015. M2 linked the payment of the cesarean section bonus to the prior reimbursement by the agency. She reviewed the contents of the cesarean section kit and removed antibiotics, pain relief and antimalarials in 2016. The number of other consumables like needles were halved. As a result, the kit completeness index (double gray line in Figure 3) dropped sharply from 95% in 2015 to 38% in 2016. The managers of the hospital felt they had strong arguments to introduce these reforms, but expressed being constrained in their autonomy:

“*If drugs are delivered for free, the pharmacy will need to be closed and patients will have to buy them in private pharmacies or will have to be referred to other hospitals. (But) we do not feel free (…) We are not comfortable at all [to take measures that safeguard financial survival of the facility but that introduce charges to users]. The status of a public hospital limits the autonomy of taking certain decisions.” (Semi-structured interview, Hospital manager, 2018)*

Despite being aware of these changes, the implementing agency did not officially notify the hospital nor sanction it. The agency managers reported that since they were failing to deliver on a critical part of the contract (for instance the timely reimbursement), they felt weakened in their capacity to enforce the policy or the contracts. They also felt it was beyond their core duties and means.

#### The ICAMO Configurations for the Period 2016–2018

The reimbursement became irregular and unpredictable. The Agency still focused on checking the expense claims but did no longer monitor how the hospitals adapted to the financial constraints induced by the reimbursement delays (Intervention). The political support for the policy became very low (Context). The national health insurance policy created competition (Context) and obtained support among both political and technical actors. The outcome was reduced implementation of the policy at the central level (O).

At the hospital, the bottom-up pressure on the hospital managers decreased from 2016 onwards (Context). The reimbursement delays (Intervention) increased the financial burden (Intermediate outcome), which the hospital managers perceived as a threat (Mechanism). Managers perceived enough autonomy (Context) to reduce the benefit package (Intermediate outcome) in order to secure the financial survival of the hospital despite fears of administrative sanctions. The latter did not materialize as neither the Agency nor the Ministry of Health felt entitled to sanction in case a party failed to implement the policy (Context). In term of outcomes, the financing for the policy reduced significantly at central level. At peripheral level, the resources allocated to the policy by hospital managers were equally reduced. Trust among the stakeholders declined.

### The Refined Programme Theory

**Figure 4:**
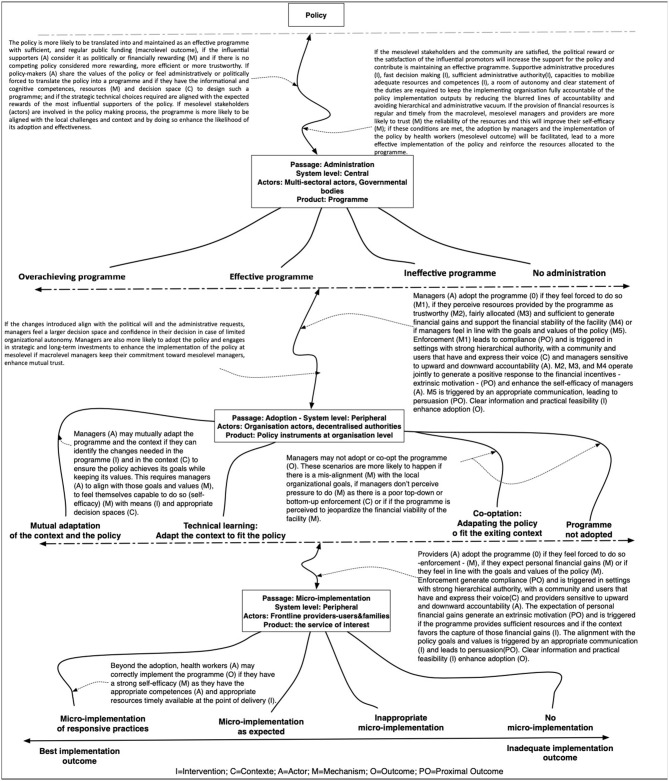
The refined programme theory.

We present the refined programme theory in a graphical format (Figure 4).

## Discussion

This study followed a policy from its inception in 2009 to 2018. It focused on the relationship between the implementing agency at central level and an implementing hospital at peripheral level. It identified two phases in the policy implementation. In a first period, both the central and peripheral level allocated sufficient resources to correctly implement the policy, while in the second period, the opposite happened.

We found that the key contextual difference was the political support and the perception of the relevance, effectiveness and sustainability of the policy as compared to available policy alternatives. We found thus that the actual implementation of a policy in the form of a programme at the central level depends on how it is perceived and endorsed by political and technical stakeholders, which influences the level of resource allocation within the public resource allocation arena.

Most of the pioneering implementation literature starts from the premise that politics determines policy goals and that implementers translate it into action (11). We found that the implementers and the final beneficiaries determine the policy implementation output as much as the policymakers. Our study shows that the objectives and expectations of policymakers concerning a given policy may change over time, influenced by (in)formal feedback from various communities experiencing the policy; such feedback shapes next phases of the policy implementation through a set of mechanisms like loss of trust, perceived inefficiency, the resurgence of ideological resistance, loss of political support, all resulting in a change of the resources allocated to the policy.

“Insufficient resources” and “opposition within the policy community” are often reported as determinants of policy implementation since the first generation of policy implementation research (12, 43). They are also mentioned in the recent literature on implementation of user fee exemption policy in sub-Saharan Africa (44–46). Funding for the policy is likely to reduce if influential stakeholders no longer support it, either because they perceive it as inefficient or untrustworthy. We found that policy community members interpret political support in terms of expected political rewards (mechanism) that may originate from the implementation of the policy. The political reward is more likely to be low if key stakeholders, including the citizens, are not satisfied (voter dissatisfaction—mechanism). If the policy implementation is hampered for instance because of inadequate central funding, the policy community may start perceiving it as a threat and disengage from it, setting off a negative feedback loop.

At the level of the hospitals, we found the vision of the managers, their perception of the policy and its instruments, the pressure they feel (either administrative and hierarchical or political in nature), the room of maneuver they perceive, and the level of trust to be determinants of policy implementation, in line with (47). We found, for instance, that one manager developed a response centered on community expectations and health workers' motivation, while the other focused on containing unnecessary cesarean sections and reducing the loss of revenue. In both cases, the manager perceived sufficient managerial autonomy to make adaptations. The timely availability of resources at the peripheral level is an intermediate outcome and a logical determinant of successful policy implementation. The timely provision of resources to the hospital improves the central level's legitimacy, contributes to higher levels of trust between local managers and their staff and lowers the stress levels of local managers as it improves their self-efficacy and allows them to plan and develop strategies. The contract introduced in 2013 did little to improve the implementation because the implementing agency failed to deliver on its own commitments, and by doing so lost the political legitimacy to enforce it. This resulted in a *laissez-faire* response from the central level to the adaptations introduced in policy implementation by local managers, even if they prevent the policy to achieve its goals.

We can also draw some methodological lessons, which we summarized in Box 1. In our study, we analyzed the behavior of the implementing agency and a hospital implementing the policy over a period of 10 years. This long period allowed for a configurational comparative analysis over time. In applying the realist evaluation approach to the study of the implementation of a policy over a long period of time, we encountered the well-known challenge of exploring policy processes that not only occurred a long time ago, but which were also not well-documented. This made developing thick descriptions (48) of the context, the policy, the process, and the actors not an easy task. Since realists seek to explore the causal pathways underlying change and thus must look for mechanisms, the task of retrospective policy analysis was even more difficult. In response, we collected data from a multitude of sources and analyzed these using different strategies. After lengthy discussions amongst the authors, we presented our preliminary analysis to key respondents, using the realist interview technique to seek respondent validation (49, 50).

Box 1 **Summary of key methodological lessons for using realist methodologies in policy implementation research.**

**What do you need? Strong policy process documentation is necessary**

Applying realist methodologies to policies may require documenting in detail key features of the policy over a long period of time. Policy process documentation is thus paramount. It has to focus on key components like policy content, policy context, policy process and policy actors.

**Where to start? Anchor the data collection process on the outcome**

Anchoring the data collection process on the outcome, meaning that we assessed the outcomes first and organized the rest of the study to explain this outcome, facilitated the study of a fluid policy process that plays out in an open system. As the potential outcome scenarios are limited, starting from there facilitates the identification of the ICAMO configurations.

**How to deal with the unexpected outcomes and conditions inherent to health policies and health systems? Engage the stakeholders, use multiple tools, technics and sources, and adopt a long-term approach**

In applying realist methodologies to policies studies, it may be challenging to define the outcome, since the policy process may have multiple (un)intended or (un)expected outputs. Another challenge is to identify unexpected context conditions that may influence the intervention, or its outputs and outcomes. Clarifying the research question or ensure a proper engagement of various stakeholders who will use the result of the research help to address those challenges. Moreover, using multiple tools, technics and sources, and adopt a long-term follow-up approach enhance the researcher to remain open and to capture unexpected relevant factors and unexpected outcomes.

**What key added values of realist methodologies in HPSR from this study? Facilitate the development of prospective policy analysis and systemic learning**

Realist evaluation can strengthen prospective policy analysis. With a configurational analytical approach centered on actors in contexts, generative causation and a focus on theory, realist evaluation can enhance the methodological ground, the feasibility and the relevance of the prospective policy analysis. It can facilitate the systematic learning by aligning realist cycles with learning cycles within the policy process.

**Where are more methodological research and guidance needed? Scientific abstraction and scientific concretisation**

Applying realist methodologies to policy studies requires a process of navigating between “scientific abstraction and scientific concretisation.” There is still little methodological guidance in the literature on how to do this. We call for more attention and guidance on this methodological process in the current debate on realist research.

“Anchoring” the data collection process on the outcome, meaning that we assessed the outcomes first and organized the rest of the study to explain this outcome, facilitated the study of a fluid policy process that plays out in an open system. As the potential outcome scenarios are limited, starting from there facilitates the identification of the ICAMO configurations.

Another methodological lesson relates to the “abstraction process” inherent to the application of the realist methodology to policy processes. There is in the current literature little guidance on this process of navigating between “scientific abstraction and scientific concretisation” (51, 52), while it is key in the methodological process. “Effective programme, for instance, is an abstract concept that requires concrete criteria of assessment. The criteria may vary in function of the policy under investigation. In our study, we used the ratio “financial resources allocated in the budget of the Ministry of Health over the number of the cesarean section performed in the accredited facilities” to make concrete the abstract concept of “effective programme.” During such analysis, the researcher shifts from the collected data to a more abstract level to refine the programme theory. We call for more attention and guidance on this methodological process in the current debate on realist research.

We also found that realist evaluation can strengthen prospective policy analysis, which is a “*real-time documentation and analysis of political economy factors and feedback to engender reform*” (53). With a configurational analytical approach centered on actors in contexts, generative causation and a focus on theory, realist evaluation can enhance the methodological ground, the feasibility and the relevance of the prospective policy analysis. It can facilitate the systematic learning by aligning realist cycles with learning cycles within the policy process. This can happen by making explicit the assumptions of policy makers and implementers and testing them prospectively (54). The refined theories which result from such studies can inform the subsequent stages of the policy process and strengthen the learning system necessary for stronger and more resilient health systems (55).

Finally, “outcome” in realist evaluation is a construct that reflects “*the practical effects produced by causal mechanisms being triggered in a given context.”* (56), which can be assessed in terms of intensity and sub-groups (57). In our study, a particular challenge was the definition of the outcome, since the policy process may have multiple (un)intended or (un)expected outputs. Another challenge is to identify unexpected context conditions that may influence the intervention, or its outputs and outcomes. Thanks to the diversity of the tools, the variety of technics and of the sources, and the long-term follow-up we felt better equipped to remain open and to capture unexpected factors and unexpected outcomes.

## Conclusion

With our study, we set out to test whether realist methodologies can be applied to policy research. In the example presented in this paper, realist evaluation made it possible to formulate plausible explanatory theories on what drives change in policy implementation outputs over time in a low-income country. At central level, the availability of resources for a given policy is constantly challenged in a competitive resource allocation arena where the political power and legitimacy of the group supporting a policy are key. Political power and legitimacy are influenced by the trustworthiness of the policy, its implementation structure and how its outputs are perceived by the community. The role of the health facilities at the meso-level, where the services are delivered, is key in this regard and depends on the trustworthiness of the resources received from the central level, the organizational autonomy, the personal attributes of the managers, and the accountability arrangements that surround the operational management of the policy.

Realist methodologies have strong potential for health policy research and especially in prospective policy analysis. This is maybe one of the way policy research can have a transformative impact necessary to foster the achievement of Sustainable Development Goals in LMICs.

## Data Availability Statement

The raw data supporting the conclusions of this article will be made available by the authors, without undue reservation.

## Ethics Statement

The studies involving human participants were reviewed and approved by Comité Local d'Éthique pour la Recherche Biomédicale, Université de Parakou (CLERB-UP), Bénin. The patients/participants provided their written informed consent to participate in this study.

## Author Contributions

J-PD, BM, and SV wrote the protocol of the study. J-PD collected the data, performed the primary analysis, drafted the first version of the manuscript, and integrated and inputs of all authors. SV and BM reviewed it extensively for inputs. All authors approved the last version.

## Conflict of Interest

The authors declare that the research was conducted in the absence of any commercial or financial relationships that could be construed as a potential conflict of interest.
